# Blockage of longitudinal flow in Meniere's disease: A human temporal bone study

**DOI:** 10.3109/00016489.2010.532155

**Published:** 2011-02-14

**Authors:** Shigetaka Shimizu, Sebahattin Cureoglu, Shigetoshi Yoda, Mamoru Suzuki, Michael M. Paparella

**Affiliations:** 1 Department of Otolaryngology, Tokyo Medical University, Tokyo, Japan; 2 Department of Otolaryngology, University of Minnesota, Minneapolis, MN, USA; 3 Department of Otolaryngology, Kawasaki Medical School, Kurashiki, Japan; 4 Paparella Ear Head and Neck Institute, Minneapolis, MN, USA

**Keywords:** Histopathology, endolymphatic hydrops, endolymphatic duct, utriculo-endolymphatic valve, ductus reuniens, fistula

## Abstract

*Conclusion:* Blockage of the endolymphatic duct is a significant finding in Meniere's disease. The position of the utriculo-endolymphatic valve (UEV) and blockage of the ductus reuniens in the temporal bones were not found to be directly indicative of Meniere's disease. *Objective:* Comparison of blockage of the longitudinal flow of endolymph between ears affected by Meniere's disease and normal ears. *Methods:* We examined 21 temporal bones from 13 subjects who had Meniere's disease and 21 normal temporal bones from 12 controls. *Results:* The endolymphatic duct was blocked in five (23%) ears affected by Meniere's disease (*p* = 0.016). The utricular duct was blocked in 16 (76%) ears affected by Meniere's disease and 11 (52%) normal ears (*p* = 0.112). The saccular duct was blocked in 6 (28%) of ears affected by Meniere's disease and 16 (76%) normal ears (*p* = 0.001). The ductus reuniens was blocked in 10 (47%) ears affected by Meniere's disease and 10 (47%) normal ears (*p* = 1.000).

## Introduction

Meniere's disease is a complex condition of the inner ear, which is the most common cause of episodic vertigo combined with fluctuating hearing loss. The etiology and pathophysiology of Meniere's disease remain controversial and are not clearly understood even after a century of research. Endolymphatic hydrops was reported independently in 1938 by Hall-pike and Cairns [1] and Yamakawa [2] and has been defined as a pathologic marker in Meniere's disease. Some studies describing the various congenital or developmental anomalies that involve the longitudinal endolymphatic drainage system, which could lead to Meniere's disease, exist in the literature [3,4]. It has been reported that endolymphatic flow may usually be blocked in the endolymphatic duct, ductus reuniens, and utriculo-endolymphatic valve (UEV) [4–7]. Although some reports in the literature discuss blockage in Meniere's disease, to the best of our knowledge, no study yet has compared temporal bones affected by Meniere's disease and normal temporal bones. Here, we report the histological findings of 42 temporal bones − 21 of those affected with Meniere's disease and 21 normal ears.

## Material and methods

We examined 21 temporal bones from 13 patients who had Meniere's disease (mean age, 68.0 years) and 21 normal temporal bones from 12 control subjects (mean age, 61.5 years). Of the former group, five ears were affected unilaterally and eight bilaterally. Clinical diagnosis of Meniere's disease was established on the basis of the criteria of the Committee on Hearing and Equilibrium, American Academy of Otolaryngology-Head and Neck Surgery.

All temporal bones were removed at autopsy, fixed in formalin, decalcified, and embedded in celloidin. Each bone was cut into serial horizontal sections of 20 mm thickness. Every 10th section was stained with hematoxylin and eosin and mounted on a glass slide for light microscopic study. We checked for blockages of the endolymphatic duct, utricular duct, saccular duct, and cochlear duct and for fistulae involving the cochlear duct, saccule, and utricle. We studied the serial sections around the continuous fistulae and excluded slides that had discontinuous fistulae.

For statistical analysis, we used two-sample *t* tests to evaluate the frequency of blockages in the different groups. A difference was considered significant if *p* < 0.05.

## Results

**Figure 1:**
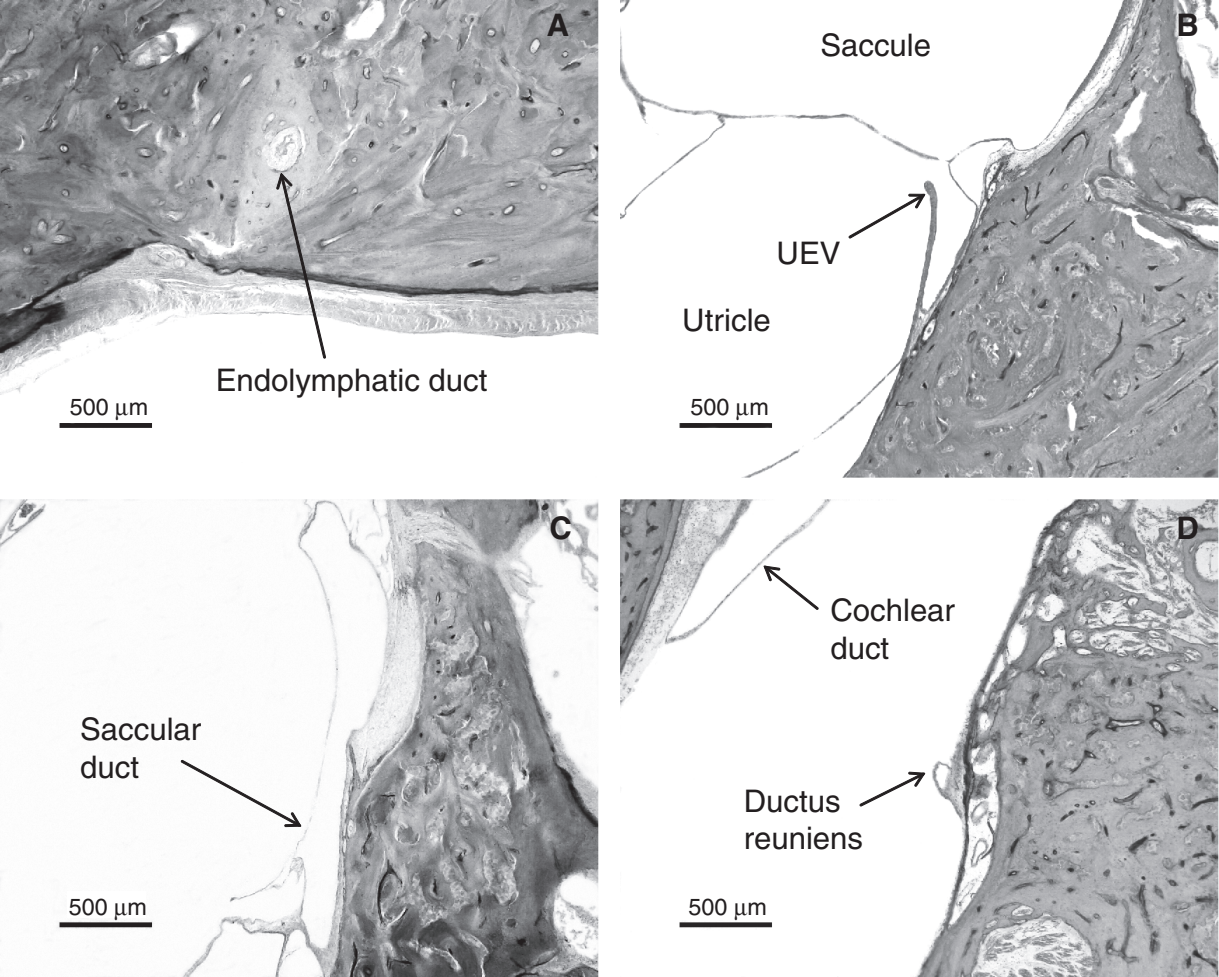
Patency of endolymph in ears affected by Meniere's disease. (A) Blockage of the endolymphatic duct. (B) Open utriculo-endolymphatic valve. (C) Open saccular duct. (D) Open ductus reuniens.

**Figure 2:**
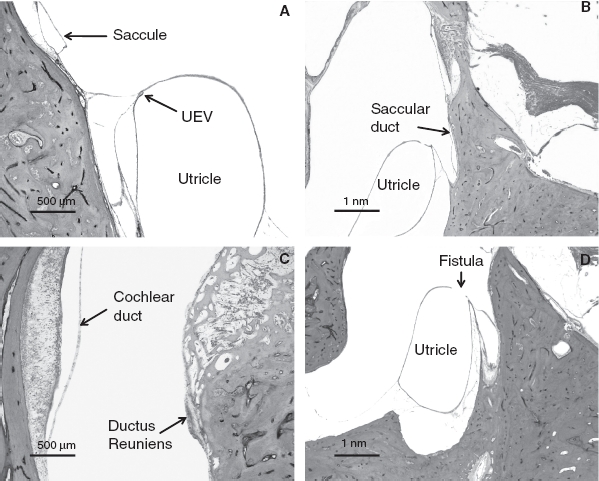
Blockages or fistulae in normal ears. (A) Closed utriculo-endolymphatic valve. (B) Collapsed saccular duct. (C) Collapsed ductus reuniens. (D) Fistula between the utricle and perilymphatic space.

The patency of endolymph in ears affected by Meniere's disease is shown in Figure 1. The endolymphatic duct was blocked in 5 (23%) of the 21 temporal bones from patients who had Meniere's disease but in none of the 21 normal temporal bones (*p* = 0.016) (Table I). Although Meniere's disease was associated with otosclerosis in three ears, the endolymphatic duct was blocked in the temporal bone of only one of these. Other reasons for blockage of the endolymphatic duct were fibrous changes in three and mucosal proliferation in one of the above-mentioned ears. The endolymphatic sinus was blocked in only one ear affected by Meniere's disease. The utricular duct was blocked in 16 (76%) ears affected by Meniere's disease and 11 (52%) normal ears; almost all of these blockages were caused at the UEV (13/16 ears affected by Meniere's disease and 11/11 normal ears) (*p* > 0.05). The utricular duct blockages in the remaining 3 of the 16 ears were caused by compression of the distended utricle. The saccular duct was blocked in 6 (28%) ears affected by Meniere's disease and 16 (76%) normal ears. The saccular duct blockages in 4 of the 6 ears affected by Meniere's disease and in all of the 16 normal ears were caused by duct collapse; the blockages in the remaining 2 ears affected by Meniere's disease were caused by compression of the dilated saccule or cochlear duct. Saccular duct blockages were significantly more frequent in the normal ears than in the ears affected by Meniere's disease (*p* = 0.001); this blockage in the latter cases was found in only one of the five ears that also had an endolymphatic duct blockage (Table II). The ductus reuniens was not blocked in any of the normal ears with no saccular duct blockage (*p* = 0.01) (Table III) but was blocked in 10 (47%) ears affected by Meniere's disease and 10 (47%) normal ears. The blockage of the ductus reuniens was caused by the collapse of the duct in all cases. All normal ears with ductus reuniens blockage also had saccular duct blockage (*p* = 0.012). A fistula was found between the saccule and perilymphatic space in 11 (52%) ears affected by Meniere's disease and 7 (33%) normal ears (*p* = 0.222). A fistula was also found between the utricle and perilymphatic space in 9 (42%) ears affected by Meniere's disease and 15 (71%) normal ears (*p* = 0.063). Endolymphatic duct blockage was not found in any of the ears affected by Meniere's disease with fistulae involving the utricle. A fistula was found between the cochlear duct and perilymphatic space in eight (38%) ears affected by Meniere's disease and one (4%) normal ear (*p* = 0.007). In addition, fistulae between the cochlear duct and saccule, and between the saccule and utricle were found in one (4%) and four (19%) ears affected by Meniere's disease, respectively. Blockages or fistulae in normal ears are shown in Figure 2.

**Table I:** Comparison of frequency of blockages or fistulae between ears affected by Meniere's disease and normal ears.

	Blockage					Fistulae				
		
Status	E duct	E sinus	U duct	S duct	DR	S-P	U-P	C-P	C-S	S-U
Meneiere's disease (*n* = 21)	5 (23%)	1 (4%)	16 (76%)	6 (28%)	10 (47%)	11 (52%)	9 (42%)	8 (38%)	1 (4%)	4 (19%)
Normal (*n* = 21)	0 (0%)	0 (0%)	11 (52%)	16 (76%)	10 (47%)	7 (33%)	15 (71%)	1 (4%)	0 (0%)	0 (0%)
*t* test	0.016	0.323	0.112	0.001	1	0.222	0.063	0.007	0.323	0.036

E, endolymphatic; U, utricle; S, saccule; DR, ductus reuniens; C, cochlear duct.

**Table II:** Complications of blockages and fistulae in ears affected by Meniere's disease.

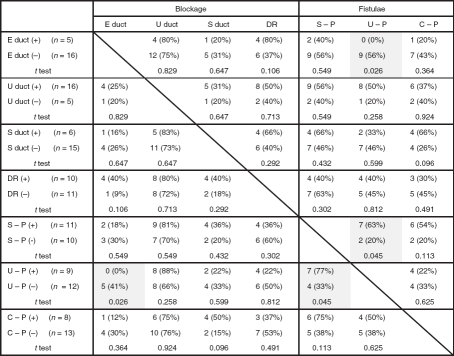

E, endolymphatic; U, utricle; S, saccule; DR, ductus reuniens; C, cochlear duct.

**Table III:** Complications of blockages and fistulae in normal ears.

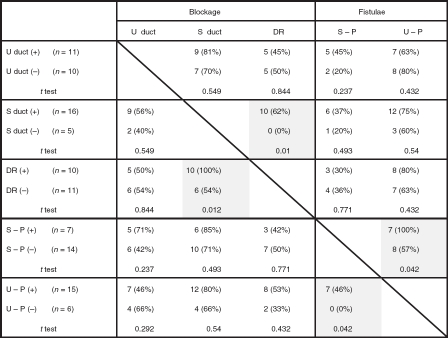

U, utricle; S, saccule; DR, ductus reuniens; C, cochlear duct.

## Discussion

The endolymphatic duct was blocked only in ears affected by Meniere's disease; this finding is consistent with the findings of the previous studies [4,8,9]. However, there was no significant difference in the frequency of utricular duct blockage between ears affected by Meniere's disease and normal ears. Schuknecht and Belal reported that the UEV is normally closed in its normal position and only became significant under pathological conditions to prevent excessive loss of endolymph from the utricle [6]. Konishi observed that the UEV opens for a few days after hydrops begins and then closes because of the compression caused by increasing hydrops [10]. In this study, the UEV was closed in 16 (76%) ears affected by Meniere's disease and 11 (52%) normal ears; the frequency of the closure was not significantly different between ears affected by Meniere's disease and normal ears. Therefore, we believe that the UEV is often closed even in normal ears under certain conditions, and the position of the UEV in the temporal bones does not directly reflect the pathological condition of Meniere's disease. The saccular duct does not have a valve like the UEV and gets blocked because it collapses on itself. The saccular duct was blocked in many normal ears but was open in ears affected by Meniere's disease and with endolymphatic duct blockage. This finding indicated that the saccular duct is usually closed to maintain the pressure within the saccule, and it opens when the pressure needs to be reduced. Blockage of the ductus reuniens is also caused by collapse. This blockage was found in 47% (10 ears) of Meniere's disease cases, which is similar to that found in 56% of cases reported by Schuknecht [4]. However, the occurrence of blockage did not differ significantly between ears affected by Meniere's disease and normal ears. This finding indicates that ductus reuniens blockage is not of great importance in the etiology of Meniere's disease. In the normal ears with open saccular ducts, the ductus reuniens was found to be open, and in normal ears with closed ductus reuniens, the saccular duct was found to be closed. These findings probably reflect the pressure of the endolymph produced in the cochlear duct. Bachor and Karmody have postulated that the collapse of the ductus reuniens is correlated to the closure of the UEV caused by decreasing pressure in the entire endolymphatic system [5]. Kitahara et al. indicated that a negative-feedback system between plasma vasopressin and its receptor in the endolymphatic sac in normal subjects could ensure inner ear fluid homeostasis [11]. These compensatory processes may be a reason for the lack of a significant relationship between the blockage of some ducts and endolymphatic hydrops in our study. The number of fistulae between the cochlear duct and perilymphatic space has been shown to be significantly higher in ears affected by Meniere's disease, and almost all fistulae were found in the Reissner membrane. Therefore, this finding would indicate the pathological condition of Meniere's disease [12–14]. However, this finding was noted only in one normal ear in this study. Further, similar numbers of fistulae between the saccule or utricle and the perilymphatic space were found in normal ears and in ears affected by Meniere's disease. Schuknecht and Rüther reported that fistulae may theoretically act as escape routes for the accumulating endolymph and thus arrest the progression of endolymphatic hydrops [4]. In fact, many fistulae involving both the saccule and utricle were found in ears affected by Meniere's disease as well as in normal ears. This finding indicates that the elevation of endolymph pressure happens in the entire inner ear. In ears with Meniere's disease and fistulae involving the utricle, the endolymphatic duct was always found to be open. The fistula involving the utricle may have arisen as a result of endolymphatic hydrops, due to hyperproduction of endolymph in the entire inner ear [15].
